# Hand-Held Echocardiography by Advanced Practice Providers in Patients with Congestive Heart Failure

**DOI:** 10.3390/jcm13020312

**Published:** 2024-01-05

**Authors:** Maria Cecilia Tagle-Cornell, Barbara S. Novais, Songnan Wen, Justin N. Shipman, Deepa R. Mandale, Andrew P. Flom, Sandeep K. Sahnan, Lindsey M. Kriz, Michelle L. Alland, Christen W. Bird, Tasneem Z. Naqvi

**Affiliations:** Division of Echocardiography, Department of Cardiovascular Medicine, Mayo Clinic Arizona, 5777 E Mayo Blvd, Phoenix, AZ 85054, USA; novais.barbara@mayo.edu (B.S.N.); wen.songnan@mayo.edu (S.W.); shipman.justin@mayo.edu (J.N.S.); mandale.deepa@mayo.edu (D.R.M.); flom.andrew@mayo.edu (A.P.F.); sahnan.sandeep@mayo.edu (S.K.S.); kriz.lindsey@mayo.edu (L.M.K.); alland.michelle@mayo.edu (M.L.A.); bird.christen@mayo.edu (C.W.B.)

**Keywords:** congestive heart failure, echocardiography, hand-held echocardiogram, heart failure, images, advanced practice providers

## Abstract

Objectives: The performed hand-held echocardiography (HHE) was evaluated and interpreted by trained advanced practice providers (APPs) on hospitalized CHF patients for image quality and interpretation by comparing with expert echocardiographer and SE findings. Background: Congestive heart failure (CHF) is associated with increased hospital admissions and mortality. While a standard echocardiogram (SE) is the gold standard for cardiac assessment, it is not readily available. Hospitalized CHF patients require rapid assessment for expedited treatment. Methods: Over 6 months, five trained APPs performed HHE on hospitalized CHF patients and interpreted: (a) left ventricular (LV) size, (b) LV ejection fraction (LVEF), and (c) right atrial pressure (RAP). The study echocardiographer reviewed and blindly interpreted the HHE images and compared them with APPs and SE findings. Kappa statistics determined the degree of agreement between APPs and the study echocardiographer’s interpretation of the HHE images and SE. Results: A total of 80 CHF patients (age 73 ± 14 years, 58% males; LVEF (by SE) 45 ± 19%; 36.3% body mass indexes ≥ 30 kg/m^2^) were enrolled. HHE interpretation by APPs had a good agreement for LVEF (kappa 0.79) with the study echocardiographer and SE (kappa 0.74) and a good agreement for RAP (kappa 0.67) with the study echocardiographer. The correlation between the absolute LVEF interpretation by the study echocardiographer on HHE and SE was r = 0.88 (*p* < 0.0001). Conclusions: Trained APPs obtained diagnostic-quality HHE images and interpreted the LV function and RAP in CHF patients in good agreement with the study echocardiographer. LVEF by HHE correlated with LVEF by SE. Our study suggests trained APPs can use HHE to evaluate LVEF and RAP in CHF patients, leading to expedited and optimized treatment.

## 1. Introduction

Hand-held echocardiography (HHE) systems are increasingly being used as an extension of the stethoscope for rapid bedside diagnoses in the emergency department and intensive care units. Advances in diagnostic methods, medical treatments, and surgical interventions increase the number of patients diagnosed with heart failure and extend the lifespan of these patients, resulting in frequent and recurrent admissions for heart failure often outside working hours. Trained advanced practice providers (APPs) can not only evaluate these applications quickly and initiate treatment as soon as possible but can also reduce the workload of cardiology echo laboratories during working hours. This application will provide the opportunity to move faster in the diagnosis and treatment of such patients. Studies have shown that trained, novice non-experts, including medical students, hospitalists, and medical residents, can learn and become efficient in HHE with formal training [1,2]. Congestive heart failure (CHF) is the largest diagnosis-related group of Medicare beneficiary discharge diagnoses in the US and is associated with recurrent hospital admissions and significant mortality, imposing a significant economic burden on health care [3]. Heart failure readmission occurs in 25% of CHF patients within 30 days of discharge [4]. Left ventricular (LV) size, LV ejection fraction (LVEF), and right atrial pressure (RAP) by inferior vena cava (IVC) size and collapsibility can be readily assessed by HHE and can provide critical information to offer immediate and effective medical care to CHF patients. On post hoc analysis, one randomized study showed decreased length of stay by hospitalists using HHE to guide heart failure management [5].

HHE has been used in clinical practice for many years by cardiologists, non-cardiologists (intensivists, emergency medicine physicians, hospitalists), and non-physician providers for a quick assessment of cardiac function, pericardial effusion, and volume status. The training curriculum for HHE varies among different medical societies and organizations [6], but most agree on the basic need for didactic lectures, hands-on training, and the proper interpretation of the studies [7,8].

The role of the advanced practice provider (APP) workforce in cardiology is rapidly expanding in both outpatient and in-patient clinical settings. In a study by Gundersen et al., it was demonstrated that cardiac nurses who have received training in using HHE can assess the volume status of heart failure patients in a clinic setting. However, their evaluation focused on the pleural cavities and IVC without imaging of the heart [9]. With formal training on focused cardiac images, APPs can facilitate the efficient delivery of medical care and reduce the length of hospital stay for CHF patients utilizing HHE at the bedside. Currently, there is no published research study on the use of HHE by APPs to evaluate cardiac size, function, and RAP in hospitalized CHF patients. Our aim in this study was to evaluate if hand-held echocardiography (HHE) performed and interpreted by trained advanced practice providers (APPs) on hospitalized CHF patients has adequate image quality and interpretation by comparing against expert echocardiographer and SE findings.

To validate the effectiveness of using HHEs at the point of care by trained APPs, we measured image quality and the accuracy of interpretation collected by the APPs and compared their results to that of an expert echocardiographer and to a standard echocardiogram—based on three metrics: cardiac size, cardiac function (LVEF < or > 40%), and RAP (inferior vena cava size).

The pilot study consisted of selecting and training APPs, enrolling CHF patients, HHE image collection and interpretation—and then comparison, assessment, and analysis:

### Sample Size

A one-year monthly analysis between 2020 and 2021 revealed that 25 to 71 in-patients, under all medical services, were discharged each month with the primary diagnosis of heart failure at our center. The average length of stay was 8.1 days, with a range of 5.6–13.2 days. We targeted an average patient enrollment of up to 20 patients per month over a six-month period for the study.

This study was approved by the Institutional Review Board. The Institutional Review Board deemed APPs performing HHE as one research cohort and hospitalized CHF patients as a second research cohort. Hence, patients’ consent was obtained by two study personnel APPs previously trained in HHE. All subjects provided written consent to participate in this study.

## 2. Study Design

### 2.1. Methods

#### 2.1.1. HHE Training for APPs

In-patient general cardiology APPs, who were the first-line medical providers for assessment and management of CHF patients, were trained to use HHE by the level 3 expert echocardiographer (EE) who had 23-year experience in echocardiography including 14 years as a medical director of echocardiography laboratories. APPs’ training included didactic lectures, two simulation laboratory training sessions with both mannequin and live models, and bedside rounds. Additionally, APPs participated in a 10 h online course on HHE [10]. Five APPs were recruited and provided informed consent to participate in this study. They were all required to perform and interpret 5 HHE studies with diagnostic image quality for LV size, LVEF, and RAP before being certified by the study echocardiographer to perform HHE on study patients. Two study personnel APPs who were also trained and certified in HHE were excluded from this study per Institutional Review Board protocol.

#### 2.1.2. Patient Selection

**Inclusion Criteria:** Patients who were 18 years or older admitted at Mayo Clinic Arizona Hospital by the in-patient general cardiology services from November 2021 to April 2022 with primary diagnosis of new-onset heart failure or heart failure exacerbation either from our emergency department or from the outpatient clinic were included in the study.

**Exclusion Criteria:** Intensive care unit and hospice patients, inability to provide consent or declined study participation, end-stage renal failure who required renal replacement therapy, and dyspneic disorder due to suspected pulmonary pathology (chronic obstructive pulmonary disease, pneumonia, pulmonary embolism).

#### 2.1.3. APPs/Patient Enrollment and HHE Imaging

A total of 80 hospitalized patients with a primary diagnosis of CHF were enrolled after informed consent was obtained by APP study personnel. All patients had baseline data collected, including age, sex, body mass index, blood pressure, heart rate, and date of the first diuretic administration. HHEs were then performed and interpreted by the trained APPs who were recruited and consented to participate in the study, as described above.

#### 2.1.4. HHE Imaging Methods

##### HHE Units

Four second-generation HHE units–Vscan Extend (GE, Healthcare, Horten, Norway) introduced in 2017 were used for the study. The units are equipped with a phased array (2–5 MHz) cardiac transducer. The unit is application based with DICOM integration and WIFI and PACs connectively. The units allow digital entry of study participant study numbers and demographics. The units have gain and depth controls along with 2D and color Doppler imaging capability as well as online linear 2D measurements capability. The units allow storage of still images as well as video loops of up to three seconds.

**Figure 1:**
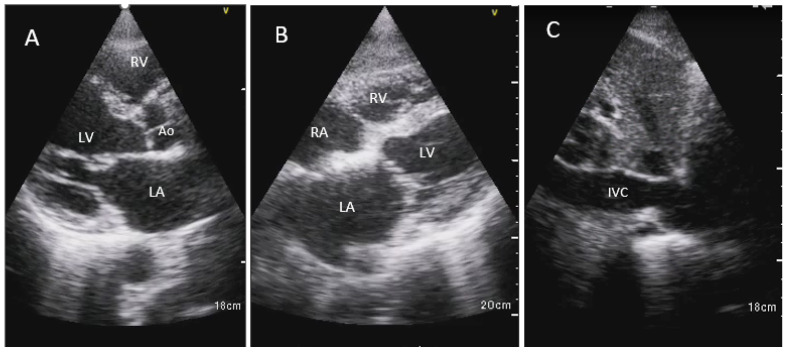
HHE images are shown in the parasternal long-axis view (**A**), subcostal 4 chamber view in the orthogonal plane (**B**), and inferior vena cava in the sagittal plane (**C**). Ao, aortic root; IVC, inferior vena cava; LA, left atrium; LV, left ventricle; RA, right atrium; RV, right ventricle.

Three views were obtained using two imaging windows: parasternal long-axis view, subcostal 4-chamber view, and IVC long-axis view (Figure 1).

RAP was estimated based on IVC diameter and inspiratory collapse as per American Society of Echocardiography recommendations [11].

APPs performed routine history and physical examination, including review of ancillary investigations such as electrocardiogram, chest radiograph, complete blood count, basic metabolic panel, NT pro-BNP, troponin, and other tests as needed for clinical care. After obtaining history and physical examination, laboratory blood work, and imaging assessment, CHF patients consented and enrolled in the study. Trained APPs then obtained HHE images on CHF patients and recorded their assessments of study quality (poor, fair, good), LV size, LVEF (< or ≥40%), and estimated RAP on a pre-printed form. HHE images were shared with the cardiology team caring for the hospitalized CHF patients and management decisions were made independent of APP interpretation of HHE images. Diuretic dose, administered route, and other cardiac medications initiated as standard of care were recorded.

##### Image Archiving

Images were saved on the HHE device. HHE units were then connected to a dedicated research desktop workstation, and study images were downloaded to a research computer.

Blinded Study Image Evaluation by Expert Echocardiographer: The EE retrieved study images on each patient blinded to the patient clinical presentation and APP assessment of image quality, LV size, LVEF, and RA pressure. Independent evaluation of all studies was performed for study quality (poor, fair, good) and assessment of LV size, LVEF, and RAP based on those images. Besides categorical cut off for LVEF of < or ≥40%, the study echocardiographer also estimated numerical % LVEF based on these images.

Non-study = related standard echocardiograms (SE) were ordered at the discretion of primary service and performed by the cardiac sonographers. The SE was interpreted by independent level 3 echocardiologists who were not aware of the research study. Comprehensive SE was performed as per clinical practice guidelines [12]. LV size, categorial LVEF at 40% cut off, and RA pressure (derived from IVC) were compared between APP interpretation and EE interpretation. In addition, numerical LVEF estimated by EE was compared against numerically calculated biplane volumetric LVEF on the SE.

#### 2.1.5. Statistical Methods

Descriptive statistics were used to summarize demographic data along with SE, HHE results, and hospitalization details. Inter-rater agreement of the evaluation of HHE imaging on LV size, LVEF, and RAP was evaluated by kappa statistics. Statistical analysis was performed using R version 3.6.2.

##### Primary Analysis between the Study Echocardiographer and APPs Assessment

Correlation of LV size, LVEF, and RAP were performed. Absolute differences in LV size, LV function, and RAP for each study were evaluated. Differences in study quality assessment were evaluated.

##### Exploratory Analysis between APPs, Study Echocardiographers, and SE

The evaluation of LV size, LVEF, and RAP between APPs and the study echocardiographer were compared against final evaluation by the SE with the expected effect of changes (especially in RAP) due to treatment by the time SE was performed.

## 3. Results

There were 80 CHF patients (age 73 ± 14 yrs., 58% male; LVEF 45 ± 19% by SE; 36% BMI ≥ 30 kg/m^2^) who were enrolled in this study. More than half (53.8%) of the hospitalized CHF patients were noted to be in atrial fibrillation on hospital admission. There were 74 patients (92.5%) whose admission diagnosis was CHF exacerbation and 37 patients (46.3%) who were diagnosed with heart failure with preserved ejection fraction (HFpEF). Hypertension (80%) was the most common co-morbidity, followed by known history of diastolic heart failure (46.3%) and smoking history (46.3%) (Table 1).

APPs recruited for this study were mostly female (80%). The highest level of education was Doctor of Nursing Practice (40%), followed by Master’s (60%). The number of years in clinical practice as an APP varied from 2 years to 16 years (Table 2).

### 3.1. Medical Therapies for CHF Patients

During hospitalization, 98.75% of the CHF patients received diuretic therapy. Only one patient did not receive a diuretic in the hospital. This patient was admitted to cardiology service based on findings of lower extremities edema and elevated NT-proBNP level from the emergency department. The patient did not appear to be clinically volume overloaded on presentation, according to the APP assessment. HHE was performed at the bedside, which revealed preserved LVEF and normal IVC with normal inspiratory collapse. These findings were subsequently confirmed with SE. She was later found to be markedly hypokalemic due to a thiazide diuretic she was taking for hypertension, which was discontinued. Potassium was repleted, and the patient was discharged home the following day.

#### Guideline-Directed Medical Therapy among Study Patients

Guideline-directed medical therapy for heart failure was optimized at the time of discharge Beta-blockers; pre-admission 49 (61.25%), at discharge 57 (73.08%), angiotensin-converting enzyme inhibitor, angiotensin renin blocker or angiotensin receptor/neprilysin inhibitor; pre-admission 48.75%, at discharge 55.13%, diuretic; pre-admission 65%, at discharge 88.46%, MRA; pre-admission 23.75%, at discharge 47.44%, SGLT2i; pre-admission 3.75%, at discharge 23.08%. For patients who could not tolerate being on angiotensin-converting enzyme inhibitor, angiotensin renin blocker or angiotensin receptor/neprilysin inhibitor due to renal dysfunction, allergies, or intolerance, hydralazine and nitrate combination were administered (pre-admission 1.25% and at discharge 3.85%) (Table 3).

**Figure 2:**
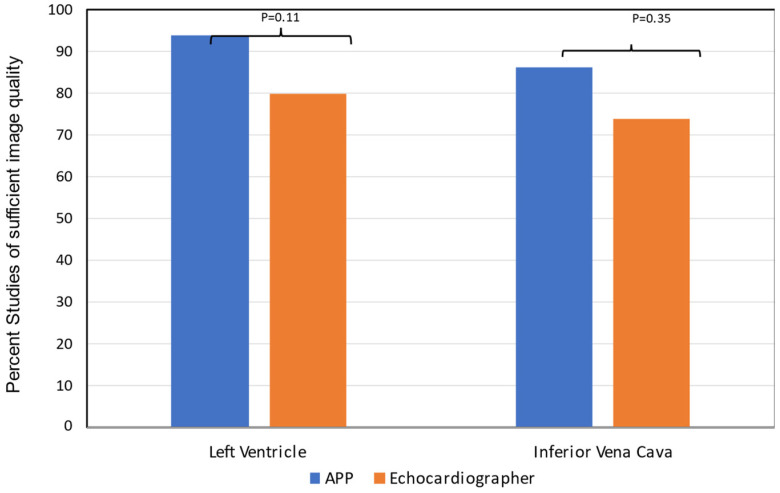
Bar graphs showing APP and study echocardiographer assessment of HHE image quality for left ventricle and inferior vena cava. Echocardiographer assessment of image quality was lower for both LV and IVC but not clinically significant.

The HHE image quality acquired by APPs was adequate to assess LV size (86%), LVEF (89%), and RAP (79%), as reviewed by the study echocardiographer (Figure 2).

On HHE, 64% of patients had LV enlargement, 51% had LVEF < 40%, and 71.4% had increased RAP, as assessed by the study echocardiographer (Table 4). LVEF (r = 0.79) and RAP (r = 0.67) by APPs had good agreement with the study echocardiographer (Table 4-top).

The HHE exam was performed prior to SE in 79% of the patients (median 0.3 days). LVEF assessment by APPs had good agreement (r = 0.74) with SE but not for LV size and right atrial pressure (RAP) (Table 4 bottom).

**Figure 3:**
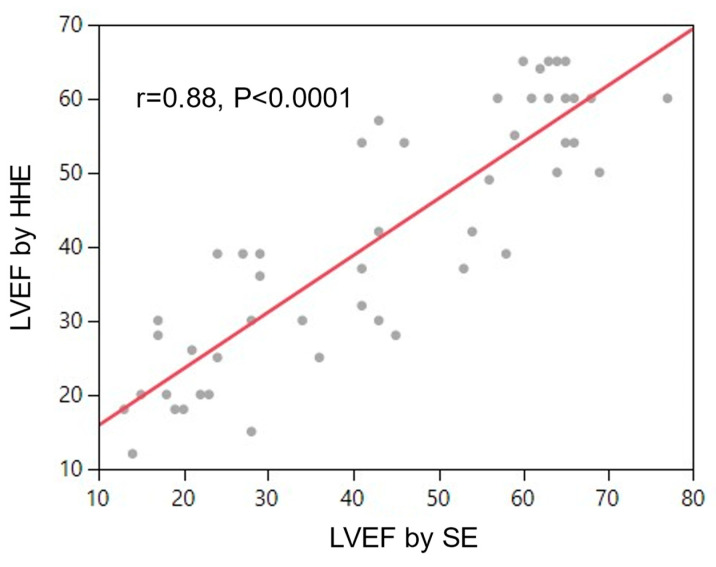
Correlation between absolute LVEF interpreted by study echocardiographer on hand-held echo images obtained by APPs vs. LVEF interpretation on standard echo by an independent echocardiographer.

Absolute LVEF on HHE as assessed by the study echocardiographer correlated with LVEF by SE (r = 0.88, *p* < 0.0001) (Figure 3).

The average length of stay for CHF patients enrolled in this study was 5.23 days compared to the historical length of stay for patients with CHF of 8.1 days immediately prior to the initiation of this study.

## 4. Discussion

APPs without prior ultrasound experience, who received dedicated HHE didactic lectures and hands-on training, were able to obtain diagnostic-quality images for the evaluation of cardiac structure, cardiac function, and volume status assessment in hospitalized CHF patients. Additionally, trained APPs demonstrated the ability to interpret their HHE findings with good correlation to an EE.

HHE image quality assessment by trained APPs was comparable to that of an EE. In addition, the HHE image interpretation of LVEF and RAP by APPs had good agreement with the interpretation of LVEF and RAP on the same images by an EE, but agreement on LV size was low. LVEF assessment at a cut off of <40% or ≥40% had good agreement (r = 0.74) with SE, but agreement for LV size and RAP was low.

Our study demonstrates that with formal training, APPs can accurately utilize HHE to obtain diagnostic quality images and interpret LVEF and RAP. Furthermore, our findings suggest that the use of HHE by APPs upon admission for CHF patients can facilitate earlier decision making, optimization of medical therapy, and potentially reduce the length of hospital stay.

It should be noted that there was a variation in RAP between SE and HHE (K value 0.39). We suspect that this may be due to the SE images being obtained after initial treatment with a diuretic for CHF. However, there was a higher agreement between the study echocardiographer and SE for RAP (K value 0.67), indicating the effect of diuretic therapy on the differences in RAP between HHE and SE to some degree.

An important observation from this study is that even if APPs are not trained to interpret LV size, LVEF at a cut off of 40%, and RA pressure assessment, they are able to obtain HHE images in the parasternal long axis and subcostal views, which can then be interpreted for the assessment of LV size, LVEF, and RA pressure by more expert echo readers. In fact, in our study, the HHE images not only showed good agreement with EE and SE on LVEF, but the quality was sufficient to allow the EE to visually quantify LVEF as a numerical value. This numerical interpretation of LVEF by EE showed an excellent correlation with biplane Simpson LVEF obtained with SE.

This study also showed an improvement in guideline-directed medical therapy for heart failure at the time of discharge, as well as a shorter average length of stay of 5.23 days compared to 8.1 days immediately prior to the initiation of the study. We did not have a control group in our study to determine if HHE reduces the length of hospital study; hence, our findings are observational. However, Lucas et al. showed that hospitalist care guided by HHE for the cardiac assessment and management of CHF patients resulted in a reduced length of stay [5,6].

Trained medical residents have been shown to be able to distinguish LVEF at a 40% cut-off range [2]. This EF cut-off range is important as it helps distinguish HFrEF from HFpEF and allows for early initiation of guideline-directed medical therapy. This is especially true for patients with presumed tachycardia-mediated cardiomyopathy due to atrial fibrillation. More than half of patients enrolled in this study were found to be in atrial fibrillation with rapid ventricular response. The presence of decreased LVEF with HHE in such patients allowed for the early restoration of sinus rhythm with chemical or electrical cardioversion.

Furthermore, evaluating cardiac function in atrial fibrillation with rapid ventricular response can pose challenges, as LVEF may appear reduced due to the fast heart rate during such episodes. However, the use of HHE for LVEF assessment, especially in patients who have undergone cardioversion (spontaneous, chemical, or electrical), can provide a relevant assessment of cardiac function in the immediate post-cardioversion setting.

Serial cardiac assessment of LV function and volume status on HHE strongly predicts readmission for heart failure patients [13]. While SE is the gold standard for cardiac assessment in such patients, it may not be readily available in the emergency department, at the time of initial admission, in late hours, and on bedside rounds. HHE can be used at initial hospital admission with suspected CHF. Its use can be expanded for patient follow up during hospital stays as part of hospital rounds for daily assessment of diuretic dose along with other heart failure medications. This is particularly useful in patients who develop an increase in their serum creatinine despite significant volume overload, which may lead to discontinuation of guideline-directed medical therapy and prolonged hospital stay.

Conducting repeated assessments of cardiac size, function, and volume status through SE in hospitalized CHF patients may not be feasible and cost efficient while also increasing the workload of already overburdened echocardiography laboratories and cardiologists. It usually takes an average of 5 min to acquire images with HHE. These images provide vital information, including LVEF, RAP, the presence of pleural and pericardial effusion, significant valvular heart disease, and aortic pathologies to make treatment decisions [14]. HHE is likely to decrease the use of SE for limited assessment of LVEF and RAP. It could potentially decrease the need for repeated radiological imaging as well as invasive hemodynamic monitoring in CHF patients. With increased use of HHE at the bedside and continued training, APPs may become more proficient with their HHE skills. Since the completion of this study, our trained APPs have routinely used HHE in hospitalized CHF patients.

APPs can also be trained on additional HHE views to identify pericardial and pleural effusions, as well as valvular abnormalities. Parasternal long axis and subcostal four-chamber views may not only allow assessment of the presence and severity of pericardial effusion but also provide 2D findings of cardiac tamponade, such as right ventricular and right atrial diastolic collapse.

There are indeed challenges that come with implementing new technology like HHE. Standardizing a training protocol for beginners and identifying models to practice on are important components of training. Additionally, there are challenges in standardizing the documentation of HHE findings in the electronic medical record, as well as image storage and billing for the use of HHE. These are all areas that need to be addressed to fully integrate HHE into healthcare systems.

Finally, our study did not formally evaluate patient attitudes towards the practice of using HHE by APPs or the effect of this intervention on patient compliance with treatment. These should be evaluated in future studies. From our clinical experience of using HHE in in-patient and outpatient practice, we have observed that both patients and their relatives find the HHE unit a significant advance in technology. Moreover, we have not encountered any resistance on the part of the patient or the family members in using this device in delivering clinical care. These images can also be used to provide direct patient education on their underlying cardiac condition at the bedside.

## 5. Limitations

There are several limitations to our study. Importantly, this study was undertaken during the COVID-19 pandemic. Alternating APP schedules with a busy hospital service limited our enrollment. Patients were also required to have the primary diagnosis of heart failure to enroll in this study. This excluded patients who were admitted with other primary diagnoses but also had heart failure exacerbation. Another limitation was that the APPs performing the HHE were not permitted (per Institutional Review Board protocol) to consent patients, so additional time was needed to identify the patients and obtain consent from non-participating APPs. Limited views were obtained, but despite this, there was good agreement on LVEF compared with SE.

We did not evaluate valvular heart disease in our study. While most HHEs do not have spectral Doppler capability, all devices allow the visual assessment of valve regurgitation severity by color Doppler, which is another parameter to follow in patients with significant valve regurgitation at the time of admission. Diastolic function and pulmonary artery pressure were also not evaluated in this study due to the lack of pulsed wave or continuous wave Doppler in the devices used. However, newer devices and software allow assessment of pulsed wave and continuous wave Doppler. Finally, our study findings are only valid in a population of heart failure patients. Future studies are needed to demonstrate APP training methods and agreement on assessment of other conditions such as pericardial effusion, valvular heart disease, and cardiac filling pressures as measurable in the newer generation devices.

### 5.1. Clinical Perspectives

This study validated the use of HHE by trained APPs in hospitalized CHF patients for assessment of LV function and RAP by IVC. Findings have implications on early recognition of cardiac function and volume status by first-line care providers (APPs) and prompt initiation of treatment in hospitalized CHF patients, which may lead to (a) early discharges and decreased length of hospital stay, (b) decreased workload burden on SE healthcare personnel, and (c) reduced healthcare costs.

### 5.2. Clinical Competencies

Given the increasing contribution of APPs to the cardiology workforce, physician shortage, and skyrocketing healthcare costs, our study findings suggest that ultrasound training and HHE use should be considered in the curriculum of APP providers via didactic lectures as well as hands-on training. The scope of practice should be defined along with quality control to maintain competency to minimize patient harm.

### 5.3. Translational Outlook

Current enhancements with artificial intelligence technologies may allow guidance for proper image acquisition by transducer positioning, as well as artificial intelligence-guided interpretation of cardiac function, filling pressures, and valve function. HHE imaging may be incorporated into the centered hospital care model.

## Figures and Tables

**Table 1:** Clinical characteristics of the study population.

Age (years)	73 ± 14
Male	48 (60.0%)
BMI (kg/m^2^)	28.5 ± 7.6
Systolic Blood Pressure	121.3 ± 17.3
Diastolic Blood Pressure	68.3 ± 12.5
Heart rate (bpm)	79.2 ± 12.4
**Admission Diagnosis**	
CHF exacerbation	74 (92.5%)
Other *	6 (7.5%)
**Discharge Diagnosis**	
CHF exacerbation	64 (80.0%)
Other *	16 (20.0%)
AF detection upon admission	43 (53.8%)
Hypertension	64 (80.0%)
iCMP	16 (20.0%)
Diabetes	20 (25.0%)
Diastolic Heart Failure	37 (46.3%)
Smoking history	37 (46.3%)
Stroke/TIA	20 (25.0%)
OSA	21 (26.3%)

Values are mean ± SD or *n* (%). AF, atrial fibrillation; iCMP, ischemic cardiomyopathy; TIA, transient ischemic attack; OSA, obstructive sleep apnea.; CHF, congestive heart failure. * Other admission diagnoses include lower extremity edema (1), weakness (1), stress cardiomyopathy (1), ischemic cardiomyopathy (1), and dyspnea (2).

**Table 2:** Clinical characteristics of the APP study population (*n* = 5).

Female	4 (80%)
**Level of Education**	
Master level	3 (60%)
Doctorate level (Doctor of Nursing Practice)	2 (40%)
**Number of Years in Clinical Practice as APP**	
1–5 years	1 (20%)
6–10 years	2 (40%)
11–15 years	1 (20%)
16 years and above	1 (20%)

**Table 3:** List of heart failure medications: pre-admission and discharge.

Medications	Pre-Admission Medications		Discharge Medications	
	*N* = 80	%	*N* = 78	%
**Beta-blockers**	49	61.25	57	73.08
**ACEi/ARB/ARNi**	39	48.75	43	55.13
**Diuretic**	52	65.00	69	88.46
**MRA**	19	23.75	37	47.44
**SGLT2i**	3	3.75	18	23.08
**Hydralazine/Nitrates**	1	1.25	3	3.85

ACEi, angiotensin-converting enzymes inhibitors; ARB, angiotensin receptor blockers; ARNI, angiotensin receptor/neprilysin inhibitor; MRA, mineralocorticoid receptor agonist’ SGLT2i, sodium-glucose cotransporter-2 inhibitors, 2 CHF pts expired in the hospital.

**Table 4:** Agreement on echocardiographic findings among APPs with the study echocardiographer and SE.

HHE Interpreted by APP vs. HHE Interpreted by EE	k-Value	95%CI
LV size: normal or enlarged	0.48	0.26–0.69
LVEF: ≥ or <40%	0.79	0.65–0.94
RAP: normal or increased	0.67	0.48–0.87
**HHE interpreted by APP vs. SE**		
LV size: normal or enlarged	0.32	0.12–0.53
LVEF: ≥ or <40%	0.74	0.59–0.89
RAP: normal or increased	0.39	0.16–0.62

EE; expert echocardiographer, LV; left ventricular; LVEF, left ventricular ejection fraction; RAP, right atrial pressure.

## Data Availability

All relevant data is included and mentioned in the manuscript.

## Abbreviations

APPs	Advanced practice providers
CHF	Congestive heart failure
EE	Expert Echocardiographer
HFpEF	Heart failure with preserved ejection fraction
HFrEF	Heart failure with reduced ejection fraction
HHE	Hand-held echocardiography
IVC	Inferior vena cava
LV	Left ventricular
LVEF	LV ejection fraction
RAP	Right atrial pressure
SE	Standard Echocardiogram
